# Palliative Care in SMA Type 1: A Prospective Multicenter French Study Based on Parents' Reports

**DOI:** 10.3389/fped.2020.00004

**Published:** 2020-02-18

**Authors:** Marie Hully, Christine Barnerias, Delphine Chabalier, Sophie Le Guen, Virginie Germa, Elodie Deladriere, Catherine Vanhulle, Jean-Marie Cuisset, Brigitte Chabrol, Claude Cances, Carole Vuillerot, Caroline Espil, Michele Mayer, Marie-Christine Nougues, Pascal Sabouraud, Jeremie Lefranc, Vincent Laugel, Francois Rivier, Ulrike Walther Louvier, Julien Durigneux, Sylvia Napuri, Catherine Sarret, Michel Renouil, Alice Masurel, Marcel-Louis Viallard, Isabelle Desguerre

**Affiliations:** ^1^Pediatric Neurology Department, Necker–Enfants Malades Hospital, APHP, Paris, France; ^2^Physical Rehabilitation Department, Necker–Enfants Malades Hospital, APHP, Paris, France; ^3^Clinical Research Department, Necker–Enfants Malades Hospital, APHP, Paris, France; ^4^Neonatal Department, Charles Nicolle Hospital, Rouen, France; ^5^Pediatric Neurology Department and Neuromuscular Diseases Reference Center, CHU, Lille, France; ^6^Pediatric Neurology Department, La Timone Hospital, APHM, Marseille, France; ^7^Pediatric Neurology Department, Enfants Hospital, Toulouse, France; ^8^Pediatric Physical Rehabilitation Department, Femme Mère Enfants Hospital, Bron, France; ^9^Pediatric Neurology Department, Pellegrin Hospital, Bordeaux, France; ^10^Pediatric Neurology Department, Armand Trousseau Hospital, APHP, Paris, France; ^11^Pediatric Department, American Memorial Hospital, Reims, France; ^12^Pediatric Neurology Department, Morvan Hospital, Brest, France; ^13^Pediatric Neurology Department, Hautepierre Hospital, Strasbourg, France; ^14^Pediatric Neurology Department & Neuromuscular Diseases Reference Center AOC, CHU Montpellier, PhyMedExp, University of Montpellier, INSERM, CNRS, Montpellier, France; ^15^Pediatric Neurology Department & Neuromuscular Diseases Reference Center AOC, CHU Montpellier, Montpellier, France; ^16^Pediatric Neurology Department, University Hospital, Angers, France; ^17^Pediatric Department, South Hospital, Rennes, France; ^18^Pediatric Department, CHU Clermont-Ferrand, Clermont-Ferrand, France; ^19^Pediatric Department, St-Pierre Hospital, Saint-Denis, France; ^20^Genetic Department, Children Hospital, CHU Dijon, Dijon, France; ^21^Palliative Care Team, Necker–Enfants Malades Hospital, APHP, Paris, France; ^22^Research Team “ETRES”, UMR des Cordeliers, Université de Paris, Paris, France

**Keywords:** SMA, palliative care, caregivers, ethics, standard of care

## Abstract

Spinal muscular atrophy type 1 (SMA-1) is a severe neurodegenerative disorder, which in the absence of curative treatment, leads to death before 1 year of age in most cases. Caring for these short-lived and severely impaired infants requires palliative management. New drugs (nusinersen) have recently been developed that may modify SMA-1 natural history and thus raise ethical concerns about the appropriate level of care for patients. The national Hospital Clinical Research Program (PHRC) called “Assessment of clinical practices of palliative care in children with Spinal Muscular Atrophy Type 1 (SMA-1)” was a multicenter prospective study conducted in France between 2012 and 2016 to report palliative practices in SMA-1 in real life through prospective caregivers' reports about their infants' management. Thirty-nine patients were included in the prospective PHRC (17 centers). We also studied retrospective data regarding management of 43 other SMA-1 patients (18 centers) over the same period, including seven treated with nusinersen, in comparison with historical data from 222 patients previously published over two periods of 10 years (1989–2009). In the latest period studied, median age at diagnosis was 3 months [0.6–10.4]. Seventy-seven patients died at a median 6 months of age[1–27]: 32% at home and 8% in an intensive care unit. Eighty-five percent of patients received enteral nutrition, some through a gastrostomy (6%). Sixteen percent had a non-invasive ventilation (NIV). Seventy-seven percent received sedative treatment at the time of death. Over time, palliative management occurred more frequently at home with increased levels of technical supportive care (enteral nutrition, oxygenotherapy, and analgesic and sedative treatments). No statistical difference was found between the prospective and retrospective patients for the last period. However, significant differences were found between patients treated with nusinersen vs. those untreated. Our data confirm that palliative care is essential in management of SMA-1 patients and that parents are extensively involved in everyday patient care. Our data suggest that nusinersen treatment was accompanied by significantly more invasive supportive care, indicating that a re-examination of standard clinical practices should explicitly consider what treatment pathways are in infants' and caregivers' best interest. This study was registered on clinicaltrials.gov under the reference NCT01862042 (https://clinicaltrials.gov/ct2/show/study/NCT01862042?cond=SMA1&rank=8).

## Introduction

Homozygous deletion of exon 7 or other mutations in the *SMN1* gene on chromosome 5q13, resulting in survival motor neuron (SMN) protein deficiency (1), causes classic proximal spinal muscular atrophy (SMA), one of the most frequently occurring neuromuscular diseases with an incidence of about 1/10,000 live births (2). SMA phenotype relies on the amount of functional SMN protein produced (3), related to the number of copies of the *SMN2* gene present in one patient (4). Distinct SMA subtypes have thus been categorized according to the age of onset and severity of the disease (5) from SMA type 0, in which onset is *in utero* with reduced or absent movements, to cases of onset in adult life (SMA type 4). The most frequent presentation remains the severe SMA type 1 [60% (6)] in which infants develop generalized progressive muscle weakness and atrophy before 6 months of age and cannot achieve independent head support nor ability to sit upright, without cognitive involvement. Associated with generalized paralysis, development of chronic respiratory failure and bulbar dysfunction in infants leads to death before 2 years of age without ventilatory support (7, 8), with recent data suggesting available simple tools to evaluate respiratory function in those infants (9). Published in 2007, the Consensus Statement for standard of Care in SMA [recently revised (10, 11)] reported different care pathways across countries and cultures, especially concerning respiratory and nutritional management (7, 8, 10–14). For the past 20 years, the French national pediatric neuromuscular network has considered a palliative care-centered approach the most ethical choice of treatment. This leads, most of the time, to an end of life before 1 year of age (7). On the other hand, in the USA, a more proactive approach with early non-invasive ventilation (NIV) and gastrostomy (GS) has been reported, leading to a more prolonged survival (12, 13, 15–17), but with an ever-increasing load of complementary and more invasive care.

In the last 5 years, drugs have been developed and reached phase I–III clinical trials (18–21): a targeted treatment has been developed with the antisense oligonucleotides (nusinersen), which alters splicing of SMN2 pre-mRNA and thus increases production of functional SMN protein. Over the last years, nusinersen has shown some clinical efficacy in well-controlled clinical trials with prolonged survival beyond 2 years of age in different populations of SMA patients, including severe SMA-1 patients (19). However, data suggest that those surviving patients require a number of technical medical supports (GS and NIV). The ethical considerations implicit in parents' and medical teams' decisions about whether or not to treat severely affected babies with nusinersen warrant a fulsome consideration of the complementary supportive care which then needs to be provided and could also modify our medical practice.

We thus intended to evaluate the evolution of our practice in palliative care, before the nusinersen era, with the active collaboration of parents, as compared with French historical data. We thus performed a prospective multicentric study over 4 years (2012–2016) about palliative care in newly diagnosed SMA-1 patients, including for the first time parents' prospective reports about care given to their child in real time until death. Parents were thus asked to give their own evaluation of the care and treatments provided to their child by a medical team, with insights on the quality of life for their child and themselves.

## Methods

From June 2012 to June 2016, patients from all pediatric neuromuscular centers in France (*n* = 17) with a genetically confirmed SMA-1 were included in a prospective study after parents signed the informed consent in accordance with national guidelines.

First, after inclusion, parents were given a specific health book (HB), developed by a multidisciplinary team that manages SMA-1 patients (physiotherapists, occupational therapists, physicians involved in neuromuscular pediatric disorders, and a pediatric palliative team). This HB contained information about the disease, advice about care (nutrition, installation, etc.), and activities adapted to infants with SMA-1. Parents were asked to fill in, at minimum every month but in fact as often as they wanted, some questionnaires about everyday care for their child. These questionnaires included both multiple-choice questions and open-ended questions requiring written free answers. The questionnaires were split into four parts: respiratory management, nutritional management, installation and physiotherapy, and aspects about pain and comfort. Parents were also encouraged to give each of their child's physician or caregiver the HB so that they could also add information about their contributions to the child's care, with specific questions for the referent physician [age at diagnosis, SMN1 deletion, number of SMN2 copies, vaccinations, weight, height, cranial and thoracic perimeters, and use of any medically related public assistance programs (medical insurance and disabled children's allowance)] and for the physiotherapist (frequency and duration of respiratory and motor interventions, use of a suction aspiration system, and physiotherapy technique used). Any other physicians and paramedics such as nurses and occupational therapists could also write any information about their involvement in that child's multidisciplinary care. After the child's death, a copy of this complete HB was obtained from the parents, and data were extracted and analyzed.

Quantitative data (responses to multiple-choice questions) were manually reported in tables using Microsoft Excel version 2010, in which medians, means, and standard deviations were then calculated. Qualitative data (open-ended questions and any comments from parents and caregivers) were manually reported, word by word, without orthographic correction, using Microsoft Word version 2010, and additional information concerning the child's care was extracted and filled in dedicated tables to enhance the accuracy of the parent-reported information. We also recorded and report which professionals wrote in the HB and the number of interventions for each child by each professional.

We also present in the article data concerning patients with SMA-1 not included in the PHRC but followed in France over the same time (retrospective study), receiving or not receiving nusinersen therapy.

Those anonymous data were retrospectively collected through the French Pediatric Neuromuscular Network: physicians of the network filled in a dedicated questionnaire containing information about age at diagnosis of SMA-1, current age or age at which death occurred, place of death, use of an enteral nutrition with or without GS, use of NIV or tracheostomy, use of analgesic or sedative medicines, and use of nusinersen therapy.

**Figure 1:**
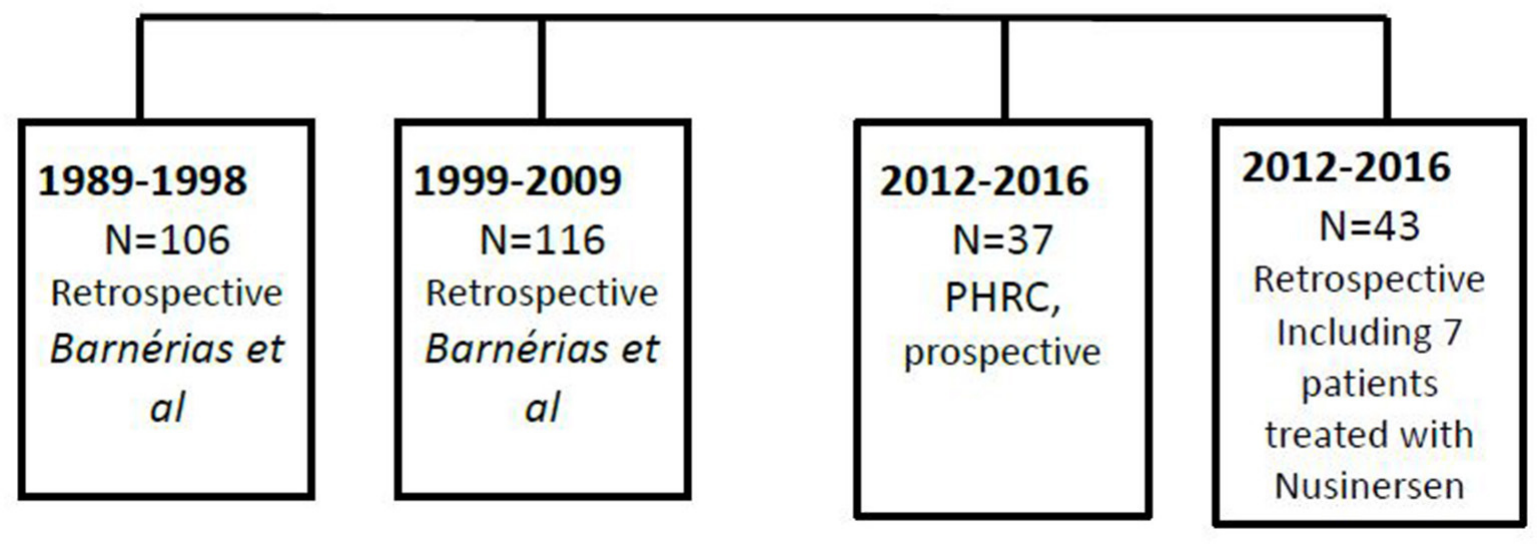
Flowchart of the patients involved in the prospective and retrospective studies (2012–2016), as well as data from previous published French study (1989–2009).

We compared our population to the historical French studies over the last 20 years. The flowchart (Figure 1) summarizes the different populations studied in the current paper.

We then compared data between prospective and retrospective studies' patients using Fisher exact tests to compare proportions and Student *t*-test or Wilcoxon test to compare means or medians, when necessary.

Because of the beginning use of nusinersen therapy during the time of the prospective study, we chose to analyze those treated patients separately and thus also compared data between patients treated or not by nusinersen using Fisher exact and Wilcoxon tests.

Statistical analyses were performed using R version 3.3.1 software.

In a second part of the study, after the child's death, the physician filled in a specific questionnaire about care and medications during the last 48 h before death, with information about possible life-sustaining treatment limitation decision and the opportunity for parents to meet a psychologist. This questionnaire contained also both multiple-choice questions and open-ended questions, which were reported and analyzed as data from the HB.

For the third part of the study, at least 6 months after the child's death, parents were encouraged to perform a semi-directed interview with a trained psychologist. Those interviews were recorded and transcribed verbatim. Then data were qualitatively analyzed via a grounded theory framework, using NVivo version 9 software. Those data are not presented in the current article but are currently processed.

This multicentric prospective French study was financed by the Ministry of Health (PHRC AOM11183) and received approval from the ethical board *Comité de Protection des Personnes* (CPP) *Ile de France II* on April 3, 2012, and registered on clinicaltrials.gov (NCT01862042).

## Results

### Population's Description

#### Patients Enrolled in the PHRC

Thirty-nine patients were included in the prospective study from 17 centers all over France during a 4-year period. Among them, two were secondarily excluded, as one patient was in fact a type 1-bis SMA, i.e., without bulbar involvement, and one was rapidly lost on follow-up. We thus report data here about only 37 SMA-1 French patients, 20 girls and 17 boys. The diagnosis was genetically confirmed, and patients were thereafter included at a median age of 3 months [0.6–10.4]. Median age reported at first signs (data available for 31 patients) was 1 month [0–4]. All the patients died, at a median age of 5.5 months [1.5–16.4], i.e., a median of 2 months [0.2–12.8] after diagnosis.

All but three HBs were retrieved, but two of them had not been filled in by the parents or the medical or paramedical therapists. We thus report the number of patients for whom the information was available for each item in our description of the population.

Table 1 presents global care used for the patients (involved in the PHRC and others over the same period of time) and reports the results available from the previous retrospective published French study (7).

**Table 1:** Presentation of the population and care use in the current prospective study as compared to the previous retrospective published French data over 20 years from Barnérias et al.

	**1989–1998 (*n* = 106)**	**1999–2009 (*n* = 116)**	**2012–2016**			
			**Prospective (*n* = 37)**	**Retrospective (*n* = 43)**	***p* (test)**	**Total (*n* = 80)**
Median age at first signs	NA	2 m [0–5]	1 m [0–4]	NA		NA
Median age at diagnostic	3 m [0.5–7]	4 m [0.5–8]	3 m [0.6–10]	3 m [2–6] (*n* = 6)	0.95 (Wilcoxon)	3 m [0.6–10.4]
Motor physiotherapy	NA	109 (90%)	26/30 (87%)	NA		NA
Respiratory physiotherapy	NA	111 (93%)	29/32 (91%)	NA		NA
Enteral feeding	36 (34%)	59 (52%)	34/37 (92%)	34/43 (79%)	0.13 (Fisher)	68/80 (85%)
Gastrostomy	2 (1.8%)	4 (3.4%)	1/37 (3%)	4/43 (9%)	0.37 (Fisher)	5/80 (6%)
Installation	28 (26%)	63 (61%)	18/20 (90%)	NA		NA
Suction aspiration system	44 (41%)	65 (64%)	25/28 (89%)	NA		NA
Oxygenotherapy	NA	10 (8%)	21/27 (78%)	NA		NA
Non-invasive ventilation at home	0	8 (7%)	4/37 (11%)	9/43 (21%), 1 tracheo	0.30 (Fisher)	13/80 (16%, 1 tracheo)
			1/37 (1%)	9/43 (21%), 1 tracheo	**0.01** (Fisher)	10/80 (1 tracheo, 12.5%)
Home hospitalization setting	35 (33%)	30 (26%)	16/26 (62%)	NA		NA
Pediatric palliative care team	NA	11%	17/23 (74%)	NA		NA
Home health nurse	NA	NA	18/24 (75%)	NA		NA
Median age at death	6 m [1–13]	7.5 m [1–24]	5.5 m [1.5–16.4]	6 m [1–27]	0.15 (*t*-test)	6 m [1–27]
Use of sedation and analgesia at the time of death	15 (18%)	65 (60%)	30/37 (81%)	27/37 (73%)	0.58 (Fisher)	57/74 (77%)

We summarized for the current study the availability of the information, as reported in the health book by parents and caregivers. Tracheo, tracheostomy.

*Bold value represents statistically significant values*.

#### Comparison Between Patients Involved in the Prospective Study and Other French Patients Over the Same Period (Retrospective Study)

We collected retrospective data concerning 43 more patients with SMA-1 not included in the prospective study presented above but followed in France (18 centers) over the same period of time. Among those patients, seven received nusinersen intrathecal therapy. At the time of the study, five patients were still alive [median age 38 months (36–59)] including four receiving nusinersen. Mean age at diagnosis (3.5 months, *SD* = 1.64) was not different from that of patients presented before (3.95 months, *SD* = 2.59) (*p* = 0.95), neither was age at the time of death (8.3 months, *SD* = 5.8 vs. 6.64 months, *SD* = 3.85, *p* = 0.15). There was no statistical difference in the number of surviving infants between the two groups (*p* = 0.06).

Data regarding the place of death were also available for those patients: 10 died at home, 6 in a regional community hospital, and 22 in a university hospital, including four in intensive care units (not different from the data presented below, *p* = 0.17).

Data regarding the use of an enteral nutrition, a GS, and NIV are reported in Table 1. No statistical differences were found comparing these two populations for enteral nutrition (*p* = 0.13), GS (*p* = 0.37), and NIV (*p* = 0.30), but the use of an NIV at home was different between the prospective (*n* = 1) and the retrospective (*n* = 9 and 1 tracheostomy) studies (*p* = 0.01).

We also compared the use of sedation (use of anxiolytics and/or grade III analgesics) for those two groups of patients. Altogether, 52 patients received sedation and 22 did not: 24 patients included in the prospective study (eight did not) and 28 patients in the retrospective study (14 did not), without reaching statistical difference (*p* = 0.61). At the time of death, 57 patients were under a sedative treatment (including 30 in the prospective study and 27 in the retrospective study), 17 were not (7 vs. 10), without reaching statistical difference (*p* = 0.58).

We then compared data between patients receiving nusinersen or not. Data are presented in Table 2, where comparisons reaching statistically significant differences are bold. We found no statistical differences in the management of patents not receiving nusinersen between the prospective and the retrospective studies. On the contrary, despite a small group of patients receiving nusinersen (*n* = 7), we found statistical differences with more patients alive in the nusinersen group, more nusinersen patients with a GS (*p* = 0.004) and an NIV (*p* = 0.016) especially at home (*p* = 0.0058), and less prescription of an analgesic treatment in the same group (*p* = 0.002).

**Table 2:** Comparison of patients not receiving nusinersen between prospective and retrospective studies and comparison between patients receiving nusinersen and those not receiving nusinersen.

	**No nusinersen**				**Nusinersen**	***p* (test)**
	**Prospective study** **(*n* = 37)**	**Retrospective study (*n* = 36)**	***p* (test)**	**Total** **(*n* = 73)**	**Retrospective study** **(*n* = 7)**	
Median age at diagnosis	3 m [0.6–10]	3 m [2–6] (*n* = 6)	0.95 (Wilcoxon)	3 m [0.6–10]	NA	NA
Number of alive patients (median age)	0/37	1/36 (NA)	0.49 (Fisher)	1/73 (1%)	4/7 (57%) (38 m [36–59])	**1.1 × 10**^**−4**^ (Fisher)
Median age at death	5.5 m [1.5–16.4]	6 m [1–27]	0.25 (*t* test)	6 m [1–27]	10 m [8–16]	0.11 (Wilcoxon)
Enteral feeding	34/37 (92%)	28/36 (78%)	0.11 (Fisher)	62/73 (85%)	6/7 (86%)	1 (Fisher)
Gastrostomy	1/37 (3%)	1/36 (3%)	1 (Fisher)	2/73 (3%)	3/7 (43%)	**0.004** (Fisher)
Non-invasive ventilation (NIV)	4/37 (11%)	5/36 (14%) and 1 tracheostomy (3%)	0.60 (Fisher)	9/73 (12%) and 1 tracheostomy (1%)	4/7 (57%)	**0.016** (Fisher)
NIV at home	1/37 (1%)	5/36 (14%) and 1 tracheostomy (3%)	0.06 (Fisher)	6/73 (8%) and 1 tracheostomy (1%)	4/7 (57%)	**0.0058** (Fisher)
Sedation	24/32 (75%)	27/35 (77%)	1 (Fisher)	51/67 (76%)	1/7 (14%)	**0.002** (Fisher)
Sedation at the time of death	30/37 (81%)	26/34 (76%)	0.77 (Fisher)	56/71 (79%)	1/3 (33%)	0.13 (Fisher)

*Bold values represent statistically significant values*.

### Supportive Care Evolution, Data From a Prospective (PHRC) Study

#### Respiratory Management

Concerning respiratory management, most patients (29/32, 91%) received respiratory physiotherapy—at home in most cases (26/29, 90%), from a median age of 4 months (0.9–12.2), most of them (20/25, 80%) at least three times a week to everyday and the others once a week, each session lasting usually around 10 min. One patient was provided with a cough assist and an intermittent positive pressure breathing device; the latter was also used by two additional patients.

Most patients (25/28, 89%) had a suction aspiration system at home; their parents had been trained to use it (21/25, 84%) and used it at home (22/25, 88%).

Most patients (30/37, 81%) received oxygenotherapy during their follow-up, including seven patients for whom the information was available only at the time of death. Many parents (21/27, 78%) reported using oxygenotherapy at home starting at a median age of 5 months (1.3–16.4).

Four patients were provided with NIV during the follow-up, but only one at home at 6.6 months (he died while on NIV). For the remaining patients, NIV use was of short duration during hospitalization, including one patient who died while on NIV. In these cases, parents reported an improvement in their child's comfort while on NIV but cited increased restriction in motion, limiting the ability to play with or cradle the treated child.

**Figure 2:**
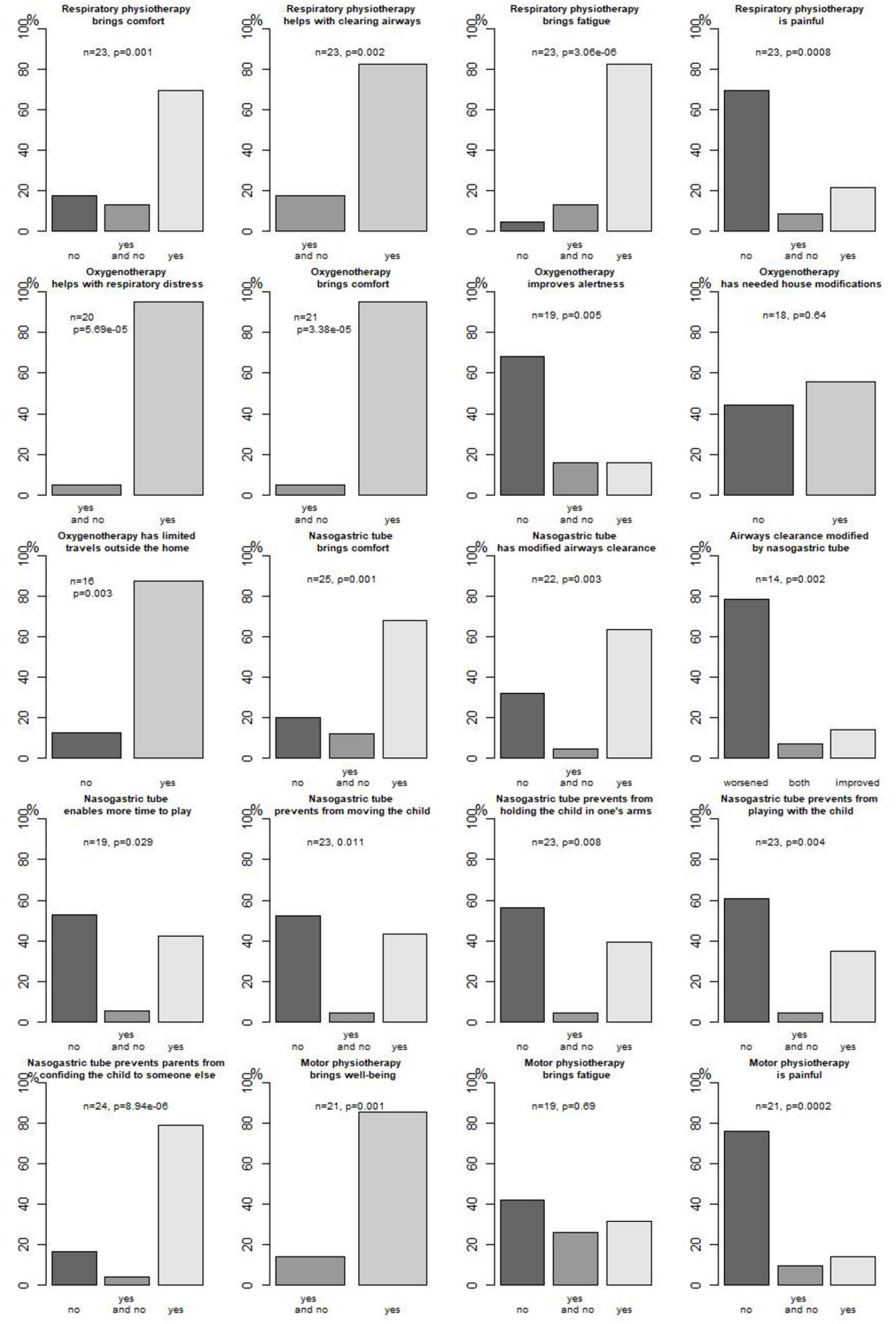
Parents' opinion about their child's technical care.

Figure 2 presents parents' opinions about respiratory management concerning oxygenotherapy and respiratory physiotherapy.

#### Nutritional Management

Most parents (27/29, 93%) reported difficulties in feeding their child at a median of 4.7 (0.8–12.2) months of age. Parents reported prolonged meal duration at a median of 6.4 months (0.8–12.2) (17/27, 63%), eating-induced fatigue at a median 5.3 months (0.8–12.2) (21/27, 78%), restricted intakes at a median of 5.3 months (1.4–12.2) (19/27, 70%), or even food refusal at a median of 5.7 months (2.4–11.8) (12/27, 44%). Parents also reported gastroesophageal reflux (GOR) in 8/27 (30%) of cases at a median age of 5.4 months (1.8–8) and food being swallowed the wrong way in 14/27 (52%) of cases at a median of 4.9 months (1.4–7.6). Constipation was also reported in 22/27 (81%) of cases.

As a result, 15/37 (41%) of patients received a treatment for GOR (six of eight patients whose parents reported GOR were treated), and 16/22 (73%) for constipation (either oral treatment or suppository or rectal enema).

Those difficulties resulted in an enteral nutrition for 34/37 (92%) of patients at a median age of 5 months (0.8–16.4), including four for whom the information was available only at the time of death. The enteral nutrition was administered through a nasogastric tube (NGT) in most cases; only one patient had a GS performed at 6.5 months of age. Enteral nutrition was begun in hospital for most (24/26, 92%) patients, and 23/26 (88%) of parents were taught how to use it, either in a hospital (20/23, 87%) or at home. While on enteral nutrition, children were delivered either homemade food (20/24, 83%) or ready-to-use therapeutic food (13/23, 57%).

Parents' opinion about enteral feeding through an NGT is presented in Figure 2.

#### Installation and Motor Management

Of 30 patients, 26 (78%) received motor physiotherapy since a median age of 3.8 months (0.9–12.2); 21/25 (84%) patients received it at home. Different techniques were used, mostly performing (11/12, 92%) postures. Parents' opinion about motor physiotherapy is presented in Figure 2.

#### Pain and Comfort Management

When reported (*n* = 27), all parents described their child as “comfortable” at a median age of 4 months (0.8–11.8). Among them, three parents first reported their child as comfortable at a median age of 4.2 months (2.5–4.4), but then as not comfortable at a median age of 5.2 months (3.5–6.8).

**Figure 3:**
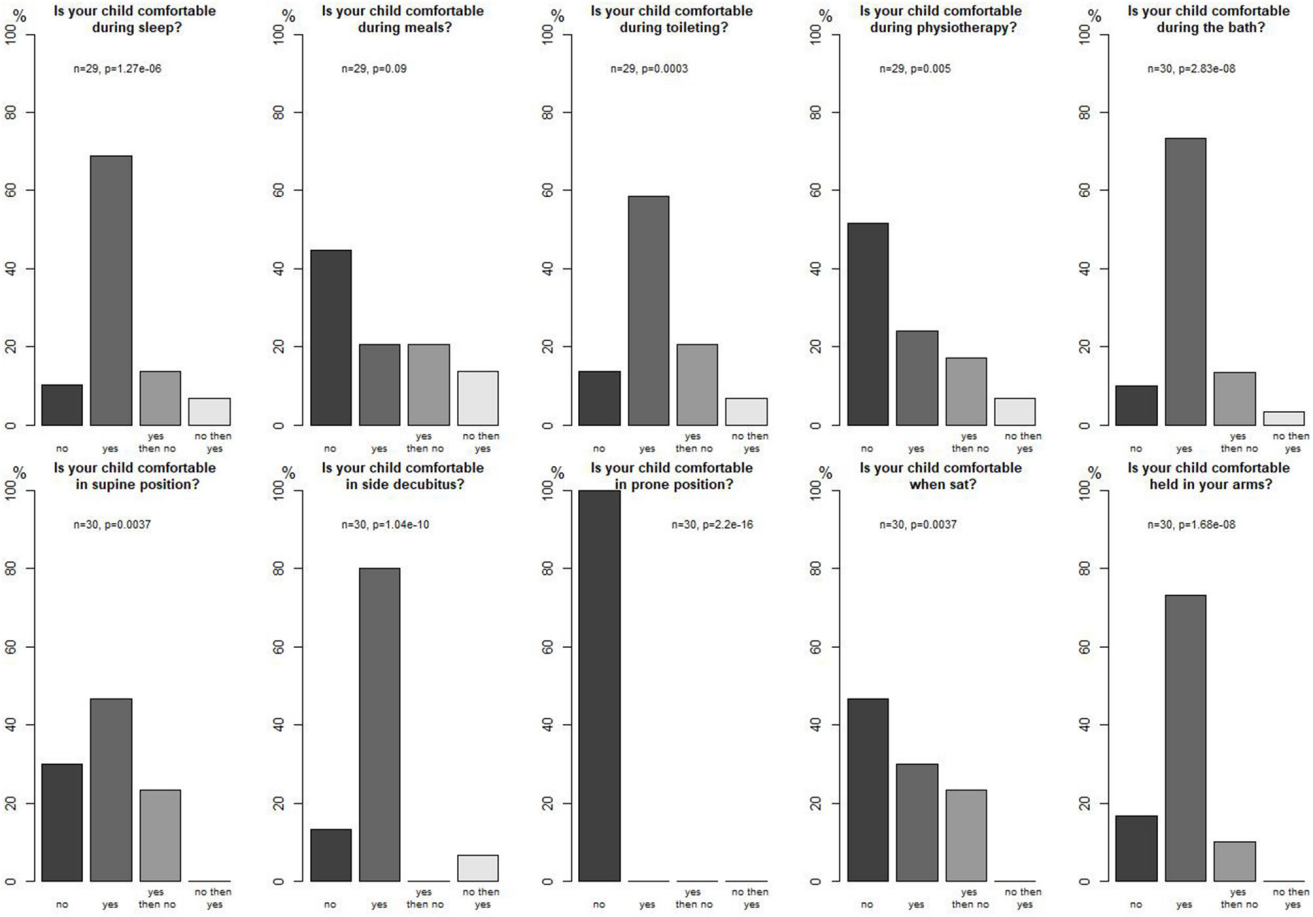
Parents' opinion about their child's comfort.

Parents evaluated their child's comfort during different activities and in different positions (significant results, *p* < 0.05), reported in Figure 3 with most patients being comfortable in side decubitus and no patient being comfortable in a prone position for instance. They also mentioned that their child seemed comfortable during strolls (*n* = 2), during hugs (*n* = 7), at play (*n* = 5), and while placed in adapted equipment (*n* = 4).

Of 33 patients, 28 (85%) reported using medicine(s) to ensure their child's comfort at a median age of 4.3 months (0.7–11.2). Those medicines included grade I analgesics (mostly paracetamol) in 19/31 (61%) since a median age of 5.2 months (0.7–12.2), grade III analgesics (mostly oral morphine) in 11/32 (34%) since a median age of 5.2 months (1.8–7.9), benzodiazepines in 8/31 (26%) since a median age of 6 months (1.8–7.9), and amitriptyline in 10/32 (31%) since 5 months of age (2–9). Parents also mentioned here the use of transdermic scopolamine in 6/31 (19%) at a median age of 7 months (5.6–9), hydroxyzine in 2 patients, and antibiotics for pulmonary infections in 11.

### Care at the Time of Death, Data From the PHRC Study

We analyzed data from a specific questionnaire about care and medications during the last 48 h before death filled in by the physician in charge of the infant, after the child's death (*n* = 37). Most patients (31/37, 84%) died because of chronic respiratory insufficiency, the others because of abrupt bulbar dysfunction leading to cardiac arrest (5/37, 14%), and for one patient, the physician was not able to identify one or another of the mechanisms. Fourteen (38%) of the 37 patients died at home, and 17 (46%) in the local university hospital (where the neuromuscular center is located) including two patients who died in the intensive care unit, and one in a public space.

Of 37 patients, 22 (59%) received grade I analgesics (paracetamol) at the time of death, and 21 (57%) received grade III analgesics (morphine), which were associated with grade I analgesics in 11 cases. Five (14%) did not receive any conventional analgesics. Fifteen (41%) patients received benzodiazepines (mostly midazolam) at the time of death, associated with grade III analgesics for 10 (27%) of them. Fifteen (41%) received amitriptyline at the time of death, one received ketamine, two (5%) received hydroxyzine, and two (5%) received scopolamine. Altogether, seven (19%) patients did not receive any sedation or grade III analgesics, and three (8%) patients did not receive any analgesic or sedative treatment (even grade I analgesic) at the time of death. Medicines were given through an NGT in most patients (31/37, 84%).

During the last 48 h before death, two patients were under NIV (2/37, 5%), 28 (28/37, 76%) received oxygenotherapy (26 through nasal cannula and two through a nasobuccal mask), and 7 (7/37, 19%) patients did not receive any specific respiratory treatment. Children were monitored on clinical examination alone for 24 patients (24/37, 65%), using a pulse oximeter for five (5/37, 14%) and a cardiorespiratory electronic scope for eight (8/37, 22%).

Do-not-resuscitate anticipated decisions were discussed with the parents for all but one patient, and a written document of the final decision was available for 32 of the patients. Three additional physicians reported that the final decision had been communicated orally (no data available for the remaining patient). The decision had been taken after a collaborative multidisciplinary discussion for 28 patients and assumed by the sole physician in eight cases.

### Caregivers' Implication and Evaluation

#### Caregivers Implicated

Data concerning the professional caregivers who provided services within the patients' home were available for 28/37 (76%) patients. A nurse provided care in the patient's home in 18 cases: as the sole care provider in two cases, in association with an in-home hospitalization service for five patients, with a palliative care team for six, and in addition to an in-home hospitalization service and a palliative care team both in five cases. In-home hospitalization service was involved in 16 cases—as the sole professional service provider in three cases and in association with a palliative care team for three. A palliative care team was involved in 17 cases, three of which were the only in-home care service for the patients.

Parents were encouraged to meet with a psychologist in 36/37 (97%) cases. Twenty-six families agreed to meeting a psychologist while their child was alive, and finally, 29 of them met a psychologist immediately before or after their child's death.

No parent reported seeking the services of a homeopath, and three reported consulting an osteopath, one an acupuncturist, three a magnetizer, and two a healer.

#### Medical and Paramedical Staff's Implication in the PHRC Study

We then reported which caregivers wrote in the specific HB for each patient. A neuropediatrician wrote commentaries in 24/37 (65%) cases, a median of 3 times for each patient (1–12). A general practitioner wrote in 19/37 (51%) HBs, a median of 1 time (1–5). For six patients, another physician (pneumopediatrician, physician in the emergency department, or physician from in-home hospitalization) wrote a median of 1 time (1–2). Of 37 physiotherapists, 22 (59%) filled in their specific questionnaire with a median of 2 free-written commentaries (1–17). Occupational therapists wrote in 4/37 (11%) HBs, a median of 4 times (2–6). Nurses wrote commentaries in 14/37 (38%) cases, a median of 2 times (1–8). The palliative care team wrote a median of 3 times (1–8) for 11/37 (30%) patients. Psychologists wrote a median of 1 comment (1–5) in 6/37 (16%) cases. Lastly, isolated comments from a social worker, psychometrician, speech therapist, midwife, and head nurse were found.

#### Parents' Implication and Opinion

As mentioned before, parents evaluated oxygenotherapy, respiratory physiotherapy, and enteral feeding (Figure 2).

Of 22 parents, 16 (73%) found the information about nutrition and feeding provided in the HB helpful. Concerning constipation, 16/22 (73%) considered it uncomfortable for their child and thus modified their child's alimentation and massaged their child's abdomen in order to improve bowel function.

Of 28 parents, 22 (79%) found the information about positioning provided in the HB helpful.

Of 27 parents, 10 (37%) also evaluated their child as being in pain, 12 (44%) as being not in pain, and 5 as being not originally in pain but becoming so at a median of 4.6 months of age (1.5–7.6). In that context, most parents (18/24, 75%) found that pain was properly rated by the medical team in charge of their child, and 20/23 (87%) found their care plan relevant to their child's needs.

Sleep was considered as not disturbed in 18/28 (64%) of patients [however, they reported more than one nocturnal awakening per night in 12/18 (67%)] and disturbed in 6/28 (21%), and for 4/28 (14%), sleep became an issue (when sleep was considered disturbed, all parents reported more than one and up to more than five nocturnal awakenings per night). Nocturnal awakenings were explained by the need for a change in position (19/28, 68%), need for massage (6/28, 21%), loss of pacifier (17/28, 61%), need for a feeding (13/28, 46%), need to be held (19/28, 68%), and need for upper-airway suction (6/28, 21%).

We also reported whether parents wrote written-free answers in the dedicated questions of the HB and also reported when they made spontaneous (i.e., not solicited by a question) comments. Only six parents did not write any solicited written-free answer; others wrote solicited written-free answers a median of 9 times (0–50). Moreover, 24/37 parents wrote spontaneous comments a median of 2 times (0–61).

## Discussion

We here report on 80 infants with severe SMA-1, followed in France between 2012 and 2016. Our data confirm previous reports on natural history for this fatal disease with a median diagnosis at 3 months (range 0.6–10.4 months) and a median age at the time of death of 6 months (range 1–27) (7). This homogeneity over the last 20 years emphasizes the ethical choice that has been made by French pediatric physicians involved in neuromuscular disorders not to implement long-term ventilation (NIV or tracheostomy) for those severe patients whose motor evolution is not modified by proactive ventilation (12, 16). However, our study confirms that palliative care is an active approach involving a multidisciplinary team, and the development of palliative care in France since 2005 (Law No. 2005-370, promulgated on April 22, 2005, and Law No. 2016-87 promulgated on February 2, 2016, regarding end of life and patients' right) has led to the implication of more home-hospital settings (26–62%) and involvement of dedicated pediatric palliative care teams (11–74%) in the management of those severe patients. If there was no evolution in NIV use over the period studied, oxygenotherapy, suction aspiration system, and enteral feeding through an NGT were used in most patients, as reported in other studies (8, 15, 22, 23) and recommended in the recently revised consensus statement on standards of care in SMA (5, 10).

Our study also collected information about medical conditions at the time of death, which occurred at home for 38% of patients vs. 17 and 23% in the previous periods reported in France (7). While there are little data among studies to enable robust comparison across countries (8, 22–24), evidence as is collectively suggests that there is an increasing consideration for parents' wishes about their child's death conditions. Morphine and benzodiazepines were used more (77%) over the last period than during the two previous ones studied in France (18 and 60%, respectively), but less than that in other studies when reported (8, 22, 23), and use of analgesics and/or sedative treatments has not been ruled yet in the last standards of care (5, 10). However, pain and dyspnea were the main symptoms reported during the last 48 h in a recent retrospective study (22), underscoring the need to prioritize comfort for those infants. Of note, amitriptyline has also been used to ensure well-being and diminish anxiety due to chronic respiratory insufficiency but needs further evaluation. Do-not-resuscitate anticipated decisions had been made for all but one patient, written in most cases (89%) and after a collaborative multidisciplinary discussion (78%), which is in accordance with current French law.

The implementation of specific pediatric palliative care in the context of SMA-1 patients needs active collaboration and coordination between the different actors involved to ensure the child's and family's best quality of life. This need for coordination has recently been supported in qualitative studies (25, 26), as well as the importance of parents' input about their wishes for their child's treatments and end-of-life conditions (22, 24, 25, 27–29).

In our study, an attempt was made to cross-validate sources to ensure the best possible description and evaluation in real life for those infants. Indeed, most technical treatments were evaluated in real life by the parents, giving insights on both benefits and disadvantages of such treatments. For instance, both respiratory and motor physiotherapy, oxygenotherapy, and NGT significantly bring comfort to the infants according to most parents without major adverse events, but for instance, an NGT was considered to worsen airway (*p* = 0.002) clearance in most cases, and especially oxygenotherapy and an NGT modified family organization [house modifications (not significant), activities, and travels]. Parents also evaluated comfort, pain, and sleep, which clearly are part of quality of life for their infants. To our knowledge, there are few studies reporting parents' opinions on specific treatments in SMA-1 patients: for instance, Davis et al. reported caregivers' opinion on nutritional management, confirming high prevalence of GOR and constipation among those children (15). As many professionals are involved in the management of those children, parents as caregivers play a major role in transmitting information, ensuring a continuum of care as no home hospitalization settings enables constant nurse or medical staff presence. This empowerment of parents in their child's care has been claimed in recent studies (24, 25, 28, 29) and seems a key point to ensure the best care for the child in real life. In our study, not only did parents report as “Clinical Research Assistant” their child's symptoms and treatments in the HB, but they also spontaneously evaluated the treatments and recommendations made by care providers, and most of all they made propositions on everyday management of a child with SMA-1 (plays, installation, and feeding), enlightening that in addition to being a caregiver, they take care of their child as every parent does.

During our study occurred the phase I–III trials with nusinersen, which since its approval by the Food and Drug Administration (FDA) at the end of 2016 has led to major hopes in the community of scientists dealing with this devastating disease. However, as mentioned above, as discrepancies existed among countries concerning nutritional and mostly respiratory management for SMA-1 patients, we chose to isolate data concerning nusinersen-treated patients in our study. Despite a small number (*n* = 7) of patients included, we found major differences in medical practice for those patients in comparison with the rest of our population study (more GS, more NIV, less analgesia and sedation, and more alive patients). This need for standardization of care has been claimed (30) to ensure better controlled studied and further analysis, especially concerning nusinersen, which remains a new drug, with lacking evidence on its long-term efficacy and tolerance profile. Moreover, major issues concerning medico-economic evaluation of orphan drugs emerge (31–34), not only of the drug itself but also of the medical organization needed to ensure appropriate deliverance and monitoring, prolonged life with potential prolonged need for technical care (enteral nutrition, respiratory support, orthopedic installation, etc.) (35, 36), or, on the contrary, improvement of respiratory function with less hospitalizations, for instance (37). In that context, not only medical costs are taken into account but also social costs for families since one parent usually needs to reduce or discontinue external employment (38). So far, no treatment cures SMA-1, and if new drugs have shown benefits on respiratory function and prolonged life (19), with improvement on motor function (21) for later-onset SMA patients, long-term evolution and especially need for long-term ventilation (even non-invasive), technical care, have to be measured. Such technical support clearly has to be evaluated in real life by the day-to-day caregivers that parents are (and of course children if possible), to ensure that their benefits overcome their burden in the condition of a still motor- and respiratory-impaired child.

## Conclusion

Whereas, natural history has not evolved since 1989 in France for SMA-1 patients, improvements concerning integrated palliative supportive care have been made, enabling more coordinated medical support, as well as more well-defined implication of parents as everyday-life caregivers to their child. However, new therapies are emerging that raise hopes but also ethical issues not only about care access and drug availability in a limited medico-economic context but also and above all about defining the child's best interest. In that context, parents need to be clearly informed on the different existing options with the remaining unknowns, before they consent to any treatment option, including their mandatory implication in their child's care and evaluation.

## Data Availability Statement

The procedures carried out with the French data privacy authority (CNIL, Commission nationale de l'informatique et des libertés) do not provide for the transmission of the database, nor do the information and consent documents signed by the patients. Consultation by the editorial board or interested researchers may nevertheless be considered, subject to prior determination of the terms and conditions of such consultation and in respect for compliance with the applicable regulations.

## Ethics Statement

This multicentric prospective French study received approval from the ethical board Comité de Protection des Personnes (CPP) Ile de France II on April 3, 2012. Information and consent forms for the participation of the child and his/her parents in the study and publication of the results were included in that approval. This study was registered on clinicaltrials.gov under the reference NCT01862042.

## Author Contributions

MH was responsible for reporting the quantitative and qualitative data, analyzed them, performed the statistical analysis, and drafted and revised the manuscript for intellectual content. MH had full access to all the data in the study and takes responsibility for the integrity of the data and the accuracy of the data analysis. CB was responsible for the study conceptualization and design, collected data, and revised portions of the manuscript for intellectual content. DC was responsible for collecting and reporting qualitative and quantitative data and revised portions of the manuscript for intellectual content. SL was responsible for collecting data, managed them, and ensured the conduct of the study. VG and ED were involved in the specific health book conception and collected data. They revised portions of the manuscript for intellectual content. CVa, J-MC, BC, CC, CVu, MM, M-CN, PS, JL, VL, FR, UL, JD, SN, CS, MR, and AM collected data and revised portions of the manuscript for intellectual content. M-LV was responsible for the study conceptualization and design and revised portions of the manuscript for intellectual content. ID was responsible for the study conceptualization and design, collected data, and drafted and revised portions of the manuscript for intellectual content. All authors read and approved the final manuscript.

### Conflict of Interest

J-MC: conference participation fees paid by Biogen and AveXis. CVu: PI for Roche studies, consultancy agreements with Roche and Biogen. VL: member of the advisory board for Biogen, AveXis, and Roche. The remaining authors declare that the research was conducted in the absence of any commercial or financial relationships that could be construed as a potential conflict of interest.
